# *Polianthes tuberosa L.* Extract suppresses melanogenesis through concurrent Inhibition of cAMP/CREB and MAPK signaling pathways

**DOI:** 10.1038/s41598-026-36962-9

**Published:** 2026-01-24

**Authors:** Qiaozhen Li, Hui Zhu, Teng Jiang, Rubiao Hou, Jinhua Li, Xiaodong Yan, Jing Wang

**Affiliations:** 1 Key Laboratory of Synthetic and Biological Colloids, School of Chemical & Material Engineering, Ministry of Education, Jiangnan University, Wuxi, 214122 Jiangsu China; 2 Brightday and Dream Biotechnology Co., Ltd, Hangzhou, 310015 Zhejiang China; 3 Jiangnan Institute of Beauty Research, Wuxi, 214111 China

**Keywords:** Anti-melanogenesis, Hyperpigmentation, Mechanistic study, Oxidative stress, *Polianthes tuberosa L*. flower, Biochemistry, Cell biology, Diseases, Drug discovery, Molecular biology, Plant sciences

## Abstract

Ultraviolet (UV) radiation is a primary environmental stimulus for skin hyperpigmentation. *Polianthes tuberosa L*. (PT), rich in polyphenols and flavonoids, possesses antioxidant and anti-inflammatory properties, yet the anti-melanogenic mechanism of the PT extract (PTE) remains unexplored. Network pharmacology revealed nuclear factor erythroid-derived 2-like 2, superoxide dismutase 1, nuclear factor kappa B subunit 1, and interleukin-6 as core targets, suggesting that PTE may coordinately modulate oxidative stress and inflammation. Consistent with this prediction, PTE dose-dependently suppressed UV-induced intracellular reactive oxygen species generation in immortalized human keratinocyte cell line (HaCaT). Furthermore, PTE not only inhibited the UV-induced release of inflammatory cytokines and paracrine melanogenic factors in HaCaT cells, but also mitigated UV-induced collagen reduction in fibroblasts. In the mouse melanoma cell line B16F10, the PTE significantly suppressed melanogenesis and tyrosinase activity (*p* < 0.001). Integration of transcriptomic and proteomic data provided a complementary view of molecular regulation at both the messenger RNA and protein levels. This convergent evidence indicated that PTE acts as a concurrent inhibitor of the cyclic adenosine monophosphate (cAMP)/protein kinase A/cAMP-response element binding protein and mitogen-activated protein kinase signaling axes, as validated by quantitative polymerase chain reaction and western blot analyses. This dual inhibition led to the downregulation of microphthalmia-associated transcription factor and its downstream melanogenic enzymes. Our findings underscore the potential of PTE as a multifaceted, natural whitening agent for cosmetic applications.

**Supplementary Information:**

The online version contains supplementary material available at 10.1038/s41598-026-36962-9.

## 1. Introduction

Ultraviolet (UV) radiation is a primary environmental driver of both cutaneous pigmentation and photodamage^1^. Specifically, UVB (280–320 nm) directly stimulates melanocyte activity and promotes melanogenesis by activating key intracellular signaling pathways, thereby contributing to dyspigmentation disorders such as melasma and post-inflammatory hyperpigmentation (PIH)^2^. In contrast, longer-wavelength UVA (320–400 nm) penetrates more deeply into the dermis, where it induces oxidative stress and accelerates aging in dermal fibroblasts, leading to collagen degradation and photoaging^3^.

Melanogenesis itself is tightly regulated by a network of signaling cascades initiated at the cell surface^4^. Key upstream receptors include the melanocortin 1 receptor (MC1R) and the stem cell factor receptor (KIT)^5^. Agonist binding (e.g., α-melanocyte-stimulating hormone (α-MSH) to MC1R) triggers the cyclic adenosine monophosphate (cAMP)/protein kinase A (PKA) signaling axis, a principal driver of melanogenic transcription^6^, whereas KIT activation by its ligand, stem cell factor, primarily stimulates the mitogen-activated protein kinase (MAPK) cascade^7^. Together, these receptor-mediated events converge to activate a core intracellular signaling network. The cAMP/PKA/cAMP-response element binding protein (CREB)/microphthalmia-associated transcription factor (MITF) axis constitutes a central regulatory pathway. Upon UVB exposure or α-MSH binding, elevated intracellular cAMP levels activate PKA^8,9^. Activated PKA then phosphorylates the CREB at Ser133^9^. Phosphorylated CREB (p-CREB) translocates to the nucleus and binds the cAMP-response element within the promoter of the MITF gene, potently inducing its transcription^10^. MITF acts as the master transcriptional regulator of melanogenesis. Once expressed, MITF binds to conserved M-box and E-box motifs in the promoters of key melanogenic enzymes—including tyrosinase (TYR), TYR-related protein 1 (TYRP1), and dopachrome tautomerase (DCT/TYRP2)—and upregulates their expression, thereby directly driving melanin synthesis^11,12^. Upstream modulation by MAPK pathways adds further complexity. The MAPK cascade, comprising subfamilies such as c-Jun N-terminal kinase (JNK), p38 MAPK (p38), and extracellular regulated protein kinases (ERK), critically regulates MITF. The JNK and p38 pathways typically respond to cellular stress and enhance MITF transcriptional activity, promoting melanogenic enzyme expression^13,14^. In contrast, ERK signaling often facilitates MITF induction indirectly by promoting CREB phosphorylation^15,16^, thereby exemplifying the multi-tiered regulatory integration within the melanogenic network.

Conventional depigmenting agents such as hydroquinone and kojic acid, despite their widespread use, often show limited long-term efficacy. Moreover, they are frequently associated with adverse effects such as cutaneous irritation, allergic reactions, and potential long-term toxicity^17,18^. These drawbacks have driven the search for safer, multitarget al.ternatives from botanical sources. Botanical extracts are increasingly favored due to their rich phytochemical composition, which supports synergistic actions against multiple pathways involved in UV-induced photodamage and hyperpigmentation^19^. For instance, glabridin from *Glycyrrhiza glabra* directly inhibits TYR activity, thereby suppressing melanogenesis^20^. Similarly, epigallocatechin gallate, a key polyphenol in green tea, modulates nuclear factor kappa-B signaling while exerting antioxidant and anti-inflammatory effects^21^. Salidroside from *Rhodiola rosea* enhances cellular resistance to UV radiation and mitigates photodamage^22^. Such polypharmacological properties make these plant-derived compounds promising candidates for next-generation depigmenting therapies.

*Polianthes tuberosa* L. (PT), a traditional ornamental plant, is known to be rich in bioactive polyphenols and flavonoids, which are associated with antioxidant and anti-inflammatory properties^23–25^. However, the anti-melanogenic potential of PT extract (PTE) and its underlying molecular mechanisms remain poorly understood, particularly its regulatory effects on UVB-induced melanogenic signaling pathways. In this study, we first characterized the phytochemical profile of PTE using ultra-performance liquid chromatography coupled with Ultra Performance Liquid Chromatography-High Resolution Mass Spectrometry (UPLC-HRMS) to identify its principal bioactive constituents. We then combined this chemical profiling with in vitro cell-based assays and an integrated dual-omics approach—encompassing transcriptomics and proteomics—to comprehensively evaluate the inhibitory effect of PTE on UVB-induced melanogenesis and to elucidate its skin-whitening mechanism. These findings provide a mechanistic foundation for the potential application of PTE as a natural skin-whitening agent.

## 2. Materials and methods

### 2.1 Chemicals and reagents

PT samples were obtained from the Dounan Flower Market (Yunnan, China). Folin-Ciocalteu reagent was purchased from Meryer Chemical Technology Co., Ltd. (Shanghai, China). The immortalized human keratinocyte cell line (HaCaT) and primary human dermal fibroblasts (HDF) were obtain from Immocell Biotechnology (Xiamen, China). Mouse melanoma cell line B16F10 cell (B16F10) was obtained from BeNa Culture Collection (Beijing, China). High-glucose dulbecco’s modified eagle medium (DMEM), RPMI-1640 medium, fetal bovine serum (FBS), and 0.25% trypsin were acquired from Gibco™ (Thermo Fisher Scientific, Inc., Waltham, MA, USA). Phosphate-buffered saline (PBS) and penicillin-streptomycin solution were obtained from HyClone Laboratories, Inc. (Logan, UT, USA). 3-(4,5-Dimethylthiazol-2-yl)-2,5-diphenyltetrazolium bromide (MTT) was purchased from Sigma-Aldrich (St. Louis, MO, USA) and Triton X-100 was purchased from Beyotime Biotechnology (Shanghai, China). The RNA extraction kit, radio immunoprecipitation assay (RIPA) lysis buffer containing protease inhibitor, sodium dodecyl sulfate polyacrylamide gel electrophoresis (SDS-PAGE) reagents, polyvinylidene fluoride (PVDF) membranes, primary antibodies (against CREB, p-CREB, P38, Phospho-p38 (p-p38), JNK, Phospho-JNK (p-JNK), ERK, Phospho-ERK (p-ERK), and β-actin), HRP-labeled secondary antibodies (goat anti-rabbit/anti-mouse, 1:3000 dilution), and chemiluminescence (ECL) chemiluminescence kit were all procured from Wuhan Servicebio Technology (Wuhan, China). The PKA activity assay kit was obtained from Enzo Life Sciences, Inc. (Farmingdale, NY, USA) and cAMP immunoassay kit was obtained from Cayman Chemical (Ann Arbor, MI, USA). Total RNA extraction reagent (TRIzol) was purchased from Thermo Fisher Scientific (Waltham, MA, USA) and ABclonal mRNA-seq Library Prep Kit was purchased from ABclonal Technology (Wuhan, China). ELISA kits for detecting interleukin-1α (IL-1α), interleukin-6 (IL-6), prostaglandin E2 (PGE2), tumor necrosis factor-alpha (TNF-α), type IV, VII, and XVII collagen (Col-IV, VII, and XVII) were purchased from Shanghai Universal Biotech Co., Ltd. (Shanghai, China), while ELISA kits for α-MSH, basic fibroblast growth factor (bFGF), and endothelin-1 (ET-1) were acquired from Signalway Antibody (College Park, MD, USA). All other chemicals, unless otherwise specified, were obtained from Sinopharm Chemical Reagent (National Pharmaceutical Group Corporation, China).

### 2.2 Determination of total phenol yield

Total phenolic content was quantified by a modified Folin–Ciocalteu assay^26^. A mixture of 0.1 mL sample, 1.0 mL Folin–Ciocalteu reagent, and 1.0 mL of 10% Na_2_CO_3_ was incubated for 90 min at room temperature in a 5 mL tube. Absorbance was measured at 760 nm using a UV-Vis spectrophotometer (Shanghai Herup International Trade Co., Ltd.), and quantification was based on a gallic acid standard curve. The total phenolic yield (mg/g) was calculated as follows:

The total phenolic yield (mg/g) = ρ × N × V/M.

where ρ is the phenolic concentration (mg/mL) from the standard curve, N is the dilution factor, V is the extract volume (mL), and M is the mass of PT powder (g).

### 2.3 Single-factor experiments for PTE extraction process optimization

Fresh PT petals were rinsed with deionized water, dried at 50 °C to constant weight, ground into fine powder, and stored in light-proof aluminum foil bags. Three key operational parameters—solid-to-liquid ratio, extraction time, and extraction temperature—were selected for systematic optimization in the PTE extraction process. This selection was based on the physicochemical properties of the target phenolic constituents and fundamental principles of mass transfer:^27,28^ (1) The solid-to-liquid ratio directly determines the concentration gradient, a primary driving force for the diffusion of solutes from plant matrix into the solvent; (2) Extraction time must be optimized to balance between achieving sufficient compound release and minimizing potential prolonged exposure to conditions that might promote oxidative degradation; (3) Extraction temperature critically influences both the solubility of phenolic compounds and the viscosity of the solvent, thereby affecting diffusion rates. Importantly, as many bioactive polyphenols in PTE are thermolabile, temperature control is essential to maximize yield while preserving compound integrity. These factors are collectively recognized as pivotal determinants of extraction efficiency and final product quality in phytochemical processes.

All experimental conditions were performed with three independent biological replicates, with duplicate technical measurements for each replicate.

#### 2.3.1 Effect of solid-to-liquid ratio

A total of 5 g of petal powder was ultrasonically extracted at 60 °C for 60 min with deionized water at solid-to-liquid ratios of 1:10, 1:15, 1:20, 1:25, and 1:50 (w/v). The extracts were centrifuged (10,000 rpm, 5 min), vacuum-filtered through a 0.22 μm membrane, and analyzed for total phenolic content.

#### 2.3.2 Effect of extract time

The petal powder (5 g) was extracted with 50 mL deionized water at 60 °C under ultrasonic treatment for 30–150 min. Subsequent steps followed Sect. 2.3.1.

#### 2.3.3 Effect of extract temperature

The petal powder (5 g) was extracted with 50 mL deionized water for 60 min at temperatures ranging from 40 to 80 °C. The same workup as in Sect. 2.3.1 was applied.

### 2.4 Chemical composition determination of PTE

Chromatographic separation was performed on a Vanquish UHPLC system (Thermo Fisher Scientific, Germany) using an ACQUITY UPLC HSS T3 column (2.1 × 100 mm, 1.8 μm) held at 35 °C. The mobile phase comprised 0.1% formic acid in water (A) and 0.1% formic acid in acetonitrile (B), delivered at 0.3 mL/min under the gradient program specified in Table 1.

Mass spectrometry was conducted using a Q-Exactive HFX mass spectrometer coupled online to the UPLC. Analysis employed electrospray ionization in both positive and negative modes with spray voltages of 3800 V (ESI+) and 3500 V (ESI-). Key settings were: sheath gas, 45 arb; auxiliary gas, 20 arb; capillary temperature, 320 °C; and vaporizer temperature, 350 °C. Full-scan MS was performed at a resolution of 60,000, followed by data-dependent MS² acquisition (resolution 15,000) of the ten most intense ions.

For compound identification, raw data files were converted to mzXML format using proteoWizard, followed by peak picking, alignment, and retention time correction with XCMS software. Compounds were identified by querying an in-house traditional Chinese medicine database (Applied Protein Technolology, Shanghai, China) with mass error < 25 ppm and MS/MS spectral matching score > 0.7. Relative quantification was based on peak area intensity, with five technical replicates ensuring data reliability.

**Table 1:** Elution requirement.

Time(min)	Mobile phase A(%)	Mobile phase B(%)
Initial	95	5
3	75	25
8.5	55	45
14	5	95
17	2	98
17.2	95	5
20	95	5

### 2.5 Network Pharmacological analysis

This study predicted the potential pharmacological targets of 28 bioactive compounds in PTE, which were identified using UPLC-HRMS. Potential targets were screened through multiple pharmacological databases: the Traditional Chinese Medicine Systems Pharmacology database provided Absorption, Distribution, Metabolism, and Excretion parameters and target information; the Traditional Chinese Medicine Integrated Database supplemented component-target interactions; and the Comparative Toxicogenomics Database contributed evidence on compound-disease target associations. All retrieved targets were standardized using the UniProt database to unify protein and gene identifiers into official nomenclature. Redundant and non-human entries were removed, resulting in a refined network of PTE bioactive compounds and their corresponding targets. This dataset established a reliable foundation for subsequent network pharmacology investigations. The target prediction process adhered to established network pharmacology guidelines, and cross-validation across databases improved the accuracy and comprehensiveness of the predictions.

### 2.6 Cell cultures

HaCaT and HDF cells were cultured in DMEM medium, and B16F10 cells were maintained in RPMI-1640 medium. All media were supplemented with 10% fetal bovine serum, 100 U/mL penicillin, and 0.1 mg/mL streptomycin. Cells were incubated at 37 °C in a humidified atmosphere containing 5% CO_2_ (Bosun Medical, Shanghai, China). Upon reaching 80–90% confluence, the cells were detached using 0.25% trypsin-Ethylenediaminetetraacetic acid and passaged at a split ratio of 1:2 to 1:3 after neutralization with complete medium. All experiments were conducted using cells between passages 10 and 20 to ensure phenotypic stability and experimental consistency.

### 2.7 The inhibitory effect of PTE on PIH measured by HaCaT cells

#### 2.7.1 Cytotoxicity assessment

Cell viability of PTE-treated HaCaT cells was determined by MTT assay. After filter-sterilization through a 0.22 μm membrane, PTE was diluted in DMEM to concentrations ranging from 0.1 to 2 mg/mL. Cells were seeded in 96-well plates at 1 × 10^5^ cells/mL (0.1 mL/well), adhered for 24 h, and then treated with PTE solutions or control medium for another 24 h. Following incubation, 0.1 mL of MTT solution (0.5 mg/mL) was added per well and incubated for 4 h. The formazan crystals were dissolved in DMSO, and absorbance was measured at 490 nm. All experiments included three independent biological replicates, with six technical replicates per condition within each experiment. Viability was calculated using the formula below.

Cell viability (%) = (OD_T_/OD_B_) × 100%.

Where: OD_T_ and OD_B_ represent the average OD values of the experimental and the blank groups at 490 nm, respectively.

HaCaT cells in logarithmic phase (1 × 10^5^ cells/mL) were seeded in 96-well plates (0.1 mL/well) and cultured for 24 h. Experimental groups were exposed to UVB radiation at doses ranging from 10 to 120 mJ/cm^2^. The irradiation was performed through a thin layer of PBS after lid removal, with the UV source positioned 15 cm above the cell monolayer. Control groups received medium only without UVB exposure. After replacing the medium post-irradiation, cells were cultured for an additional 24 h, and viability was assessed using the MTT assay as described above.

#### 2.7.2 Determination of secretion of inflammatory factors (IL-1α, IL-6, PGE2, TNF- α) by ELISA

HaCaT cells were resuspended at a density of 2 × 10^5^ cells/mL and seeded in 24-well plates at 0.5 mL per well. After completing cell culture, the blank control group was treated with DMEM medium alone. Both the control group and experimental groups were irradiated with 20 mJ/cm^2^ UVB, followed by treatment with either DMEM medium or DMEM medium containing 0.05, 0.1, 0.2 mg/mL PTE. The levels of inflammatory factors (IL-1α, IL-6, PGE2, TNF-α) in the culture supernatants were measured using sandwich-based commercial ELISA kits, according to the manufacturer’s protocols.

#### 2.7.3 Determination of paracrine melanogenic factors (α-MSH, bFGF, and ET-1) by ELISA

Cell operation refers to 2.7.2. The paracrine melanogenic factors (α-MSH, bFGF, and ET-1) in the culture supernatants were quantified with specific commercial ELISA kits based on the sandwich antibody technique, in strict accordance with the manufacturer’s instructions.

Three independent biological replicates were performed for each experimental condition.

### 2.8 Measurement of intracellular reactive oxygen species (ROS)

HaCaT cells in the log-phase growth were detached using trypsin and re-suspended to a density of 2 × 10^5^ cells/mL prior to seeding into 12-well plates. Following a 24-hour period of UVB irradiation and PTE treatment, the 2’,7’-Dichlorodihydrofluorescein diacetate (DCFH-DA) probe was prepared according to the ROS detection ELISA kit protocol. Specifically, it was diluted 1:1000 in serum-free medium to achieve a final concentration of 10 µM. Each well was then incubated with 1 mL of the prepared DCFH-DA working solution at 37 °C for 20 min in darkness. Subsequently, the cells were gently rinsed three times with pre-warmed, serum-free DMEM. Fluorescence images were captured using an inverted fluorescence microscope (Motic China Group Co., Ltd., Xiamen, China). The cells were then trypsinized, re-suspended at 5 × 10^4^ cells/mL, and transferred to opaque 96-well plates for quantitative analysis. The fluorescence intensity was measured at excitation and emission wavelengths of 488 and 525 nm, respectively, using a microplate reader. All experiments were performed with three technical replicates per treatment condition, and the entire study was independently repeated three times to ensure biological reproducibility.

### 2.9 The collagen synthesis-promoting effects of PTE measured by HaCaT and HDF cells

#### 2.9.1 Cytotoxicity assessment

The effects of PTE and UVA on HDF cell viability were assessed using a protocol similar to that described in Sect. 2.7.1.

#### 2.9.2 Determination of collagen (Col-Ⅳ, Ⅶ, and ⅩⅦ) by ELISA

HDF cells were resuspended at a density of 2 × 10^5^ cells/mL and seeded in 24-well plates at 0.5 mL per well. After completing cell culture, the blank control group was treated with DMEM medium alone. Both the control group and experimental groups were irradiated with 12 J/cm^2^ UVA, followed by treatment with either DMEM medium or DMEM medium containing 0.2, 0.6, 1 mg/mL PTE.

For Col-XVII determination, HaCaT cells were treated as described in Sect. 2.7.2.

Levels of Col-IV, VII, and XVII in culture supernatants were quantified using specific commercial ELISA kits based on the sandwich antibody technique, in strict accordance with the manufacturer’s instructions. Three independent biological replicates were performed for each experimental condition.

### 2.10 Anti-melanogenic effects of PTE

#### 2.10.1 Cytotoxicity assessment

The effects of PTE and UVB on B16F10 cell viability were assessed using a protocol similar to that described in Sect. 2.7.1.

#### 2.10.2 Determination of melanin content

Melanin in B16F10 cells was quantified using a NaOH lysis method^29^. Cells were seeded at 2 × 10^5^ cells/mL in 6-well plates and cultured for 24 h. The blank group received fresh medium, while UVB and treatment groups were irradiated (20 mJ/cm^2^) and then exposed to 0, 0.2, 0.6, or 1 mg/mL PTE. After 24 h, cells were trypsinized, centrifuged, washed with PBS, and lysed in 1 M NaOH with 10% DMSO at 80 °C for 1 h. Absorbance was measured at 405 nm.

#### 2.10.3 Determination of TYR activity

TYR activity was measured using a previously described method with minor modifications^30^. Cell culture and treatment followed the procedure outlined in Sect. 2.9.2. After treatment, cells were lysed with 0.4 mL of 1% Triton X-100 in PBS at 4 °C for 30 min. The lysates were centrifuged at 12,000 rpm for 5 min, and 0.05 mL of supernatant was transferred to a 96-well plate and mixed with 0.15 mL of 10 mmol/L L-DOPA. After incubating at 37 °C for 30 min, absorbance was measured at 475 nm.

For both melanin and TYR activity assays, each experimental condition was tested in three independent biological replicates, each with three technical replicates to ensure reproducibility.

### 2.11 Transcriptome analysis

Total RNA was extracted from UVB- and PTE-treated B16F10 cells using TRIzol reagent, with three independent biological replicates per experimental condition to ensure statistical reliability. RNA quality was verified using a NanoDrop 2000 spectrophotometer (Thermo Scientific, USA) and an Agilent Bioanalyzer 4150 system (Agilent Technologies, USA), with all samples having an RNA integrity number ≥ 7.0. Sequencing libraries were constructed from ≥ 0.001 mg total RNA using the ABclonal mRNA-seq Lib Prep Kit, which included mRNA enrichment with oligo(dT) beads, fragmentation, cDNA synthesis, adapter ligation, and polymerase chain reaction (PCR) amplification. Library quality was assessed with the Agilent Bioanalyzer, and high-throughput sequencing was performed on an Illumina Novaseq 6000 or MGISEQ-T7 platform. Raw reads were quality-filtered and aligned to the reference genome. Gene expression quantification and differential expression analysis were performed using High-Throughput Sequence Analysis and Differential Expression analysis for Sequence data 2, respectively, with significance thresholds set at |log_2_FC| ≥ 1 and *P* < 0.05.

### 2.12 Proteomic analysis

This study employed LC-MS/MS-based proteomics to compare the proteomic profiles of UVB-exposed B16F10 cells treated with or without PTE. Three biological replicates per condition were analyzed. Proteins were extracted using RIPA buffer containing protease and phosphatase inhibitors, quantified by the bicinchoninic acid (BCA) assay, and verified for integrity via SDS‑PAGE.

Label‑free quantification was applied, with peptide intensities normalized against the total ion current to correct for technical variability. Proteins were digested with trypsin (1:50 enzyme‑to‑protein ratio), desalted using C18 cartridges, and separated by nano‑HPLC over a 120‑minute gradient. MS analysis was conducted in data‑dependent acquisition mode, with full scans from 350 to 1500 m/z and higher collisional dissociation fragmentation.

Data were processed with MaxQuant using the UniProt mouse database. Differentially expressed proteins (DEPs) were identified (*p* < 0.05, |log_2_FC| > 1), and subjected to functional analysis, including Gene Ontology (GO) annotation, Kyoto Encyclopedia of Genes and Genomes (KEGG) pathway enrichment, and protein–protein interaction (PPI) network construction and so on. Technical replicates were performed to ensure reproducibility.

### 2.13 Reverse transcription PCR experiments

Total RNA was extracted using a commercial RNA extraction kit, and its purity and concentration were assessed with a Nanodrop 2000 spectrophotometer. Reverse transcription was performed using a PCR system (Eastwin Life Sciences Inc, China) in a 0.02 mL reaction mixture containing 0.01 mL RNA, 0.004 mL 5× SweScript All-in-One SuperMix for quantitative PCR (qPCR), 0.001 mL gDNA Remover, and RNase-free water. Q-PCR was carried out on a Bio-Rad system with the following program: 95 °C for 30 s; 40 cycles of 95 °C for 15 s and 60 °C for 1 min; fluorescence acquisition at 0.5 °C increments; and a final step at 95 °C for 15 s. Primer sequences and target genes are listed in Table 2. All reactions were performed in triplicate, with GAPDH serving as the internal reference gene. Relative gene expression was calculated using the comparative ΔΔCt method:

A = CT (target gene, test sample) – CT (internal standard, test sample).

B = CT (test sample, control sample) – CT (internal standard, control sample).

K = A-B.

Fold change = 2^− K^.

**Table 2:** Primer design for B16F10 cells.

Gene	Primers sequence
MC1R	F: CTCATTGACGTGCTCATCTGTGG
	R: TGCTTGTAGTAGGTGATAAAGAGGGT
Kit	F: CTCTGGACCTGGATGATTTGCT
	R: GCAGTCGTGCATTTCCTTTGA
MITF	F: GCCCTATGGCTATGCTCACTCTT
	R: TGTTCATACCTGGGCACTCACTC
TYR	F: ATCCTAACTTACTCAGCCCAGCA
	R: CTCAGGTGTTCCATCGCATAAA
TYRP-1	F: TTCGTTGGAGCTGTGATTGTTG
	R: AGGAATAATGTTGAAAGGTGGGG
TYRP-2	F: CAGAAATAATGAGAAACTGCCAACC
	R: TCCGTCTGCTTTATCAAACCCT
GAPDH	F: CCTCGTCCCGTAGACAAAATG
	R: TGAGGTCAATGAAGGGGTCGT

The selected genes represent key regulators and enzymes in the melanogenic pathway. Among them, MC1R and Kit encode melanocyte-specific receptors that initiate melanogenic signaling; MITF is the master transcriptional regulator of melanogenesis; TYR, TYRP1, and TYRP2 encode the core enzymatic machinery responsible for melanogenesis.

### 2.14 Determination of activity of PKA and cAMP by ELISA

B16F10 cells were seeded in 24-well plates at 2 × 10^5^ cells/mL (0.5 mL/well) and divided after adhesion into the following groups: blank (RPMI-1640 medium only), UVB (20 mJ/cm^2^ UVB + medium), and experimental (same UVB + 0.2, 0.6, or 1 mg/mL PTE in RPMI-1640). PKA activity and cAMP levels in the culture supernatants were quantified using specific commercial ELISA kits based on the sandwich antibody technique, in strict accordance with the manufacturer’s instructions. Three independent biological replicates were performed for each experimental condition.

### 2.15 Western blotting (WB)

Cells were lysed in RIPA buffer with protease inhibitor on ice, centrifuged, and protein concentration was determined by BCA. Three independent biological replicates were performed for each experimental condition. Samples (0.02 mg) were separated by SDS-PAGE, transferred to PVDF membrane, and blocked with 5% skim milk. Membranes were incubated with primary antibodies (CREB, p-CREB, p38, p-p38, JNK, p-JNK, ERK, p-ERK, β-actin) at 4 °C overnight, followed by HRP-conjugated secondary antibody. Protein bands were visualized using an enhanced ECL detection system. To account for potential variations in protein loading, β-actin was used as an internal loading control. Band intensities were quantified using AIWBwell™ software, and the expression levels of target proteins were normalized to those of β-actin.

### 2.16 Statistical analysis

The data were analyzed by Origin software, and are expressed as mean ± standard deviation. After confirming the suitability of the data for parametric tests, one-way analysis of variance followed by Tukey’s post hoc test was applied for group comparisons. A minimum of three biological replicates were used for each experiment, ensuring statistical reliability, and a p-value of less than 0.05 was established as the threshold for statistical significance.

## 3. Results

### 3.1 optimization of extraction parameters for PTE production

**Fig. 1:**
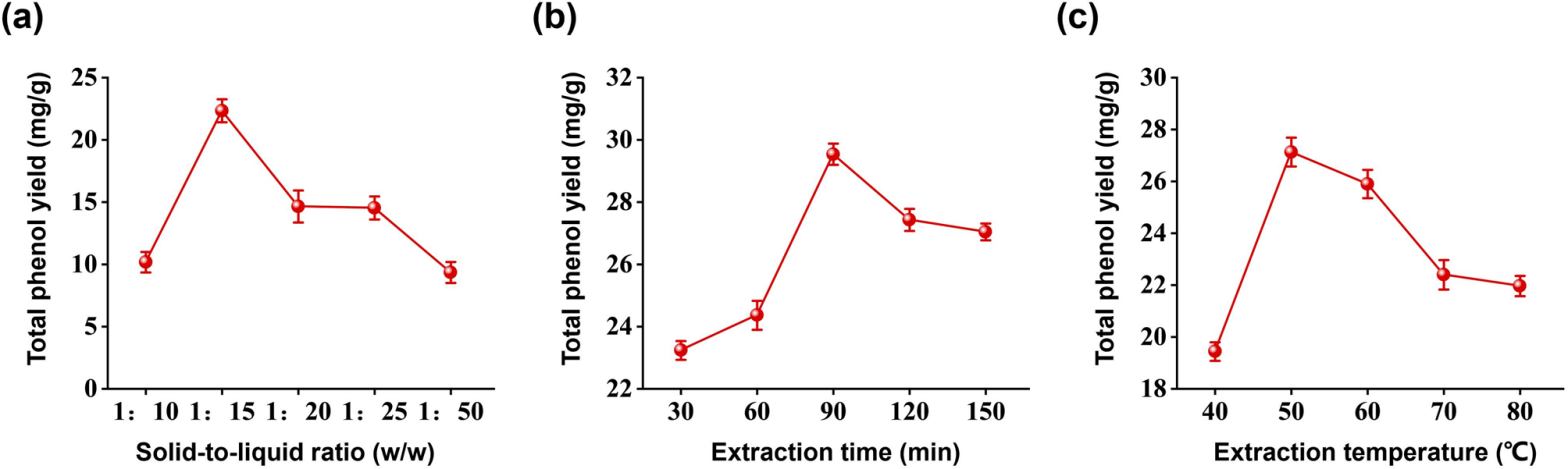
Effect of (**a**) solid-to-liquid ratio, (**b**) extraction time and (**c**) extraction temperature on the yield of total phenolics from PTE.

This study systematically investigated the effect of solid-to-liquid ratio, extraction time, and extraction temperature on the yield of total phenolic compounds (TPC) to identify optimal extraction conditions. As depicted in Fig. 1a, the TPC yield increased with solvent volume up to a solid-to-liquid ratio of 1:15 (w/v), beyond which a decline was observed. Figure 1b illustrates that the maximum TPC yield was achieved after 90 min of ultrasonication. Regarding temperature optimization (Fig. 1c), 50 °C was identified as the optimal condition, as it yielded the highest TPC content. The extraction temperature of 50 °C was selected to balance efficiency with compound preservation. While moderate heating enhances solvent diffusivity and mass transfer, promoting phenolic release^31^, excessive heat threatens thermolabile constituents like key flavonoids in PT flower, which may degrade above 60 °C^32^. Thus, 50 °C serves as a pivotal compromise: it maximizes extraction yield while safeguarding the structural integrity and bioactivity of heat-sensitive phytochemicals—a critical prerequisite for subsequent biological evaluation. Based on these results, the optimal extraction parameters were established as follows: solid-to-liquid ratio of 1:15 (w/v), extraction temperature of 50 °C, and an extraction time of 90 min under ultrasonication.

### 3.2 chemical composition determination of PTE

**Fig. 2:**
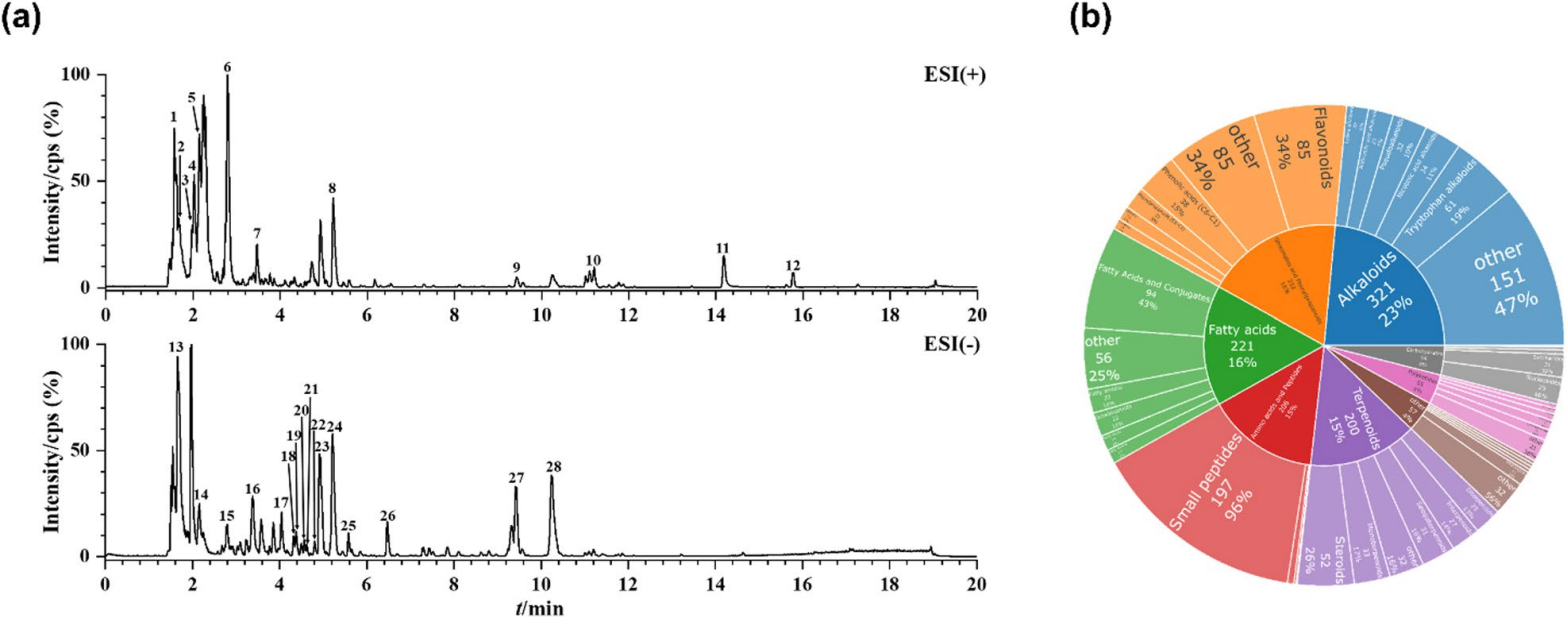
UPLC-HRMS analysis of ingredients in of PTE. (**a**) Positive ion BPC and negative ion BPC (**b**) The proportion of all identified compounds in each chemical classification.

The chemical profile of the PTE was characterized using UPLC-HRMS. Figure 2a shows the base peak chromatogram (BPC). A total of 1,495 compounds were detected, with 915 identified in positive ion mode (POS) and 642 in negative ion mode (NEG), and no overlapping compounds were observed between the two modes. According to the NPClassifier system, these compounds were classified into major categories including alkaloids (e.g., tryptamine alkaloids and pseudoalkaloids), amino acids and peptides (e.g., small peptides and oligopeptides), carbohydrates (e.g., nucleosides and sugars), fatty acids (e.g., fatty acid derivatives and eicosenoic acids), polyketides (e.g., aromatic polyketides and cycloalkanes), ceramides, phenylpropanoids (e.g., flavonoids and coumarins), and terpenes (e.g., monoterpenes and sesquiterpenes). Among these, flavonoids (85 compounds) and fatty acids (94 compounds) emerged as the predominant superclasses (Fig. 2b). The flavonoid content aligns with the typical phytochemical profile of many medicinal plants and directly explains the observed bioactivities. Flavonoids are well known for their potent antioxidant and anti-inflammatory properties, which are crucial for mitigating UV-induced skin damage and modulating melanogenesis^33^. Likewise, the abundant fatty acids and their derivatives likely contribute to skin barrier repair and exhibit anti-inflammatory effects^34,35^, potentially creating synergy with flavonoids to enhance the overall depigmenting and protective actions of PTE. The 28 major peaks on the BPC were analyzed, corresponding to compounds such as adenosine, quercetin, oleamide, and citric acid, among others (Table S1). Based on high spectral matching scores (score > 0.7), key compounds including kaempferol (a flavonoid aglycone) and oleamide (a fatty acid amide) were confirmed with high confidence. The extensive chemical diversity of PTE provides a substantial material basis for its potential bioactivities, and establishes a critical foundation for further exploration of its pharmacological properties and practical applications.

### 3.3 Network pharmacology-based prediction of active components and multi-target mechanism of PTE

**Fig. 3:**
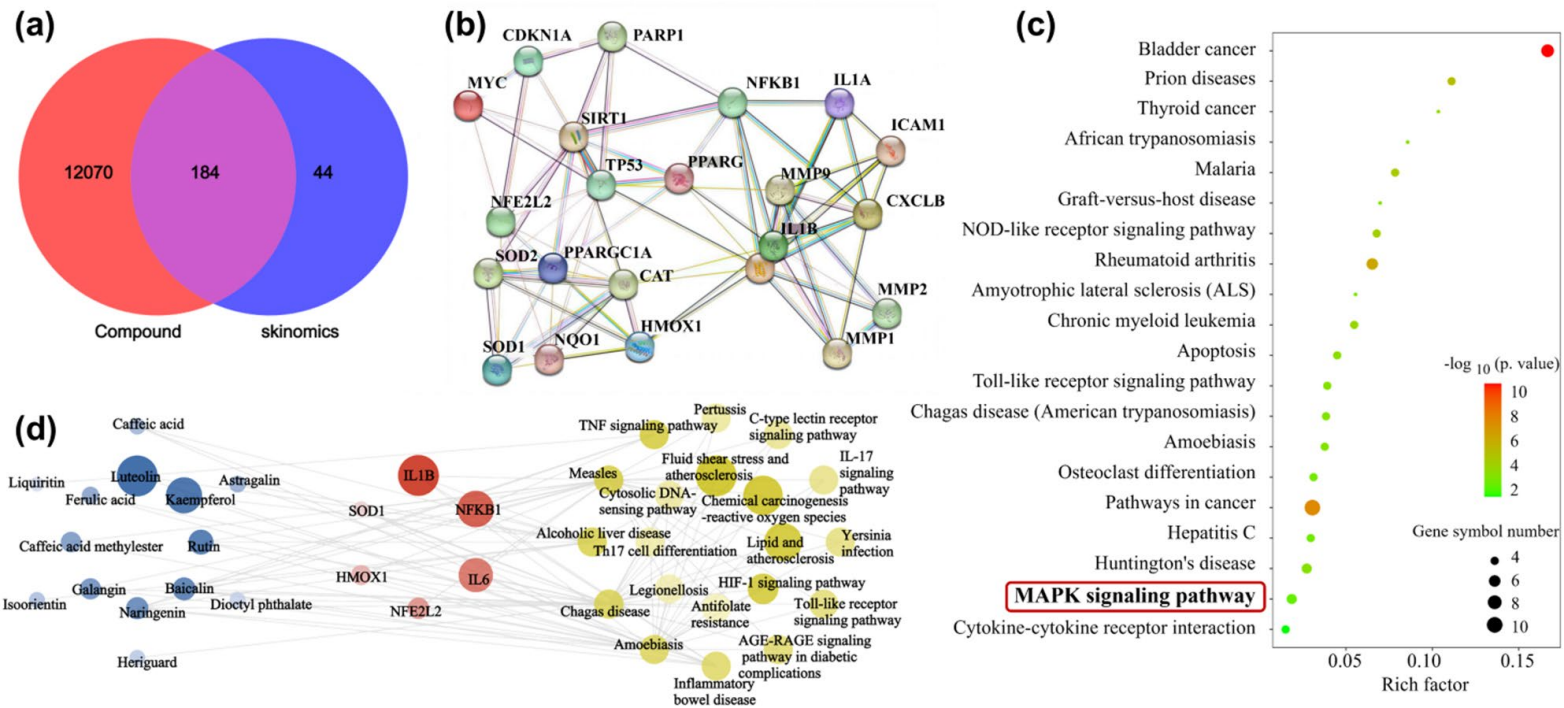
Network pharmacology-based analysis of the anti-melanogenic mechanism of PTE. (**a**) Venn diagram showing the overlap between predicted targets of PTE compounds and known targets related to skin pigmentation. (**b**) PPI network of the common targets identified in (**a**). Node size reflects the degree of connectivity. (**c**) The top 20 enriched KEGG pathways. (**d**) “Compound-Target-Pathway” network illustrating the interactions among 14 key active components, 6 core targets.

This study employed a network pharmacology approach to systematically explore the active constituents of PTE and their potential targets. Initial multi-database screening identified 12,254 potential target compounds associated with PTE, among which 228 were relevance to its efficacy. Intersection analysis revealed 184 common targets (Fig. 3a), indicating their potential critical involvement in the melanin-inhibitory activity of PTE.

PPI network was constructed using the Search Tool for the Retrieval of Interaction Gene/Proteins database, comprising 22 core nodes and 75 interactions(Figure 3b). Topological analysis identified the top 10 hub genes, including sirtuin 1 (SIRT1), peroxisome proliferative activated receptor, gamma (PPARG), PPARG coactivator 1 alpha (PPARGC1A), superoxide dismutase 1 (SOD1), superoxide dismutase 2 (SOD2), nuclear factor kappa B subunit 1 (NFKB1), nuclear factor erythroid-derived 2-like 2 (NFE2L2), myelocytomatosis oncogene (MYC), matrix metallopeptidase 2 (MMP2), and matrix metallopeptidase 9 (MMP9). By focusing on polyphenolic and flavonoid components of PTE and incorporating KEGG pathway enrichment analysis (top 20 pathways, Fig. 3c), a comprehensive “compound–core target–pathway” network was established (Fig. 3d). This network encompasses 14 key active compounds, 6 core targets (NFE2L2, IL-6, interleukin-1 beta (IL1B), SOD1, heme oxygenase 1 (HMOX1), NFKB1), and 20 major signaling pathways.

The results suggest that PTE exerts synergistic depigmenting and skin-reparative effects through the following mechanisms. (1) Antioxidant Regulation: The core targets NFE2L2 and SOD1 are key regulators of oxidative stress responses^36,37^. PTE may enhance antioxidant defenses, thereby alleviating UVB-induced oxidative damage; (2) Inhibition of Melanogenic Pathways: PTE significantly modulates the MAPK signaling pathway, potentially suppressing the expression of MITF and downstream TYR-related proteins; (3) Anti-inflammatory and Skin Barrier Repair: By targeting inflammatory mediators such as NFKB1, IL-6, and IL1B, PTE reduces UV-induced skin inflammation^38–40^, thus diminishing inflammatory stimulation of melanocytes and preventing PIH. Furthermore, the balanced regulation of MMP9 and MMP2 contributes to the maintenance of basement membrane integrity, thereby preventing photodamage-induced disruption of the dermal-epidermal junction and reducing pigment deposition^41,42^.

3.4 Inhibitory effect of PTE on UVB-induced secretion of inflammatory cytokines and paracrine melanogenic factors in HaCaT cells.

According to the MTT assay, PTE concentrations exceeding 0.2 mg/mL reduced the viability of HaCaT cells to below 80% (Figure S1a). Therefore, all subsequent experiments were conducted using PTE at concentrations ≤ 0.2 mg/mL. To establish an inflammation-induced PIH model, we evaluated the effect of varying doses of UVB irradiation (0–160 mJ/cm^2^) on the viability of HaCaT cells. The results demonstrated a dose-dependent decrease in cell viability with increasing UVB exposure (*p* < 0.01, Figure S1b). A UVB dose of 20 mJ/cm^2^ was selected for further experiments, as it resulted in approximately 70% cell viability, providing substantial photodamage while minimizing excessive cytotoxicity.

**Fig. 4:**
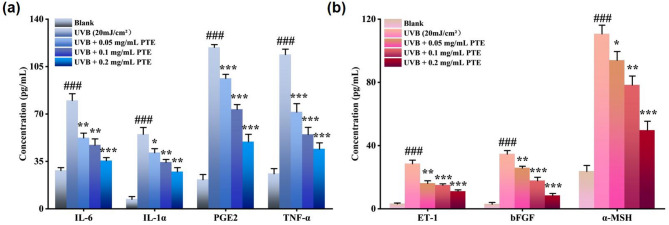
Inhibitory effect of PTE on UVB-induced factor secretion in HaCaT cells. (**a**) Secretion levels of inflammatory cytokines. (**b**) Secretion levels of paracrine melanogenic factors. *p < 0.05, **p < 0.01, ***p < 0.001 compared with UVB-treated group; #*p* < 0.05, ##*p* < 0.01, ###*p* < 0.001 compared with blank group.

To investigate the potential of PTE in mitigating PIH, HaCaT cells were irradiated with 20 mJ/cm^2^ UVB and subsequently treated with various concentrations of PTE for 24 h. The secretion levels of inflammatory cytokines (IL-1α, IL-6, PGE2, and TNF-α) and paracrine melanogenic factors (ET-1, α-MSH, and bFGF) were then measured. As shown in Fig. 4a, UVB irradiation markedly stimulated the secretion of these inflammatory cytokines, while PTE treatment suppressed their release in a dose-dependent manner.

Previous studies have indicated that under UVB-induced inflammatory conditions, keratinocytes release paracrine factors such as ET-1, α-MSH, and bFGF, which promote melanogenesis by activating corresponding receptors on melanocytes^43,44^. Consistent with these findings, UVB irradiation at 20 mJ/cm^2^ significantly upregulated the expression of α-MSH, bFGF, and ET-1 in HaCaT cells. As illustrated in Fig. 4b, PTE treatment effectively inhibited the release of these paracrine factors in a concentration-dependent manner.

To further elucidate the mechanistic basis of PTE’s anti‑inflammatory and anti‑paracrine effects, we examined its influence on oxidative stress—a pivotal upstream driver of UVB‑induced skin responses. As shown in Figure S2, UVB irradiation provoked a pronounced increase in intracellular ROS generation (*p* < 0.001), which was suppressed by PTE in a dose‑dependent manner. This antioxidant activity aligns with the network pharmacology prediction that PTE targets key regulators of oxidative stress, such as NFE2L2 and SOD1, and provides a plausible explanation for its concurrent downregulation of inflammatory cytokines and melanogenic factors. Collectively, these findings suggest that PTE alleviates UVB‑triggered pro‑pigmentary responses not only by attenuating the secretion of pro‑melanogenic paracrine factors from keratinocytes but also by mitigating the underlying oxidative stress that fuels both inflammatory and pigmentary pathways.

### 3.5 the interventional effect of PTE on UV-induced collagen degradation

To establish a model of collagen degradation, we first systematically evaluated the effect of UVA irradiation dose on the viability of HDF cells. As shown in Figure S3a, cell viability decreased significantly with increasing UVA dose. A UVA dose of 12 J/cm^2^ was selected for subsequent experiments, as it resulted in approximately 70% cell viability—sufficient to induce substantial photodamage while avoiding excessive cytotoxicity. MTT assays further revealed that PTE concentrations above 1 mg/mL significantly reduced HDF cell viability to below 80% (Figure S3b). Therefore, a PTE concentration of ≤ 1 mg/mL was used in all further treatments.

**Fig. 5:**
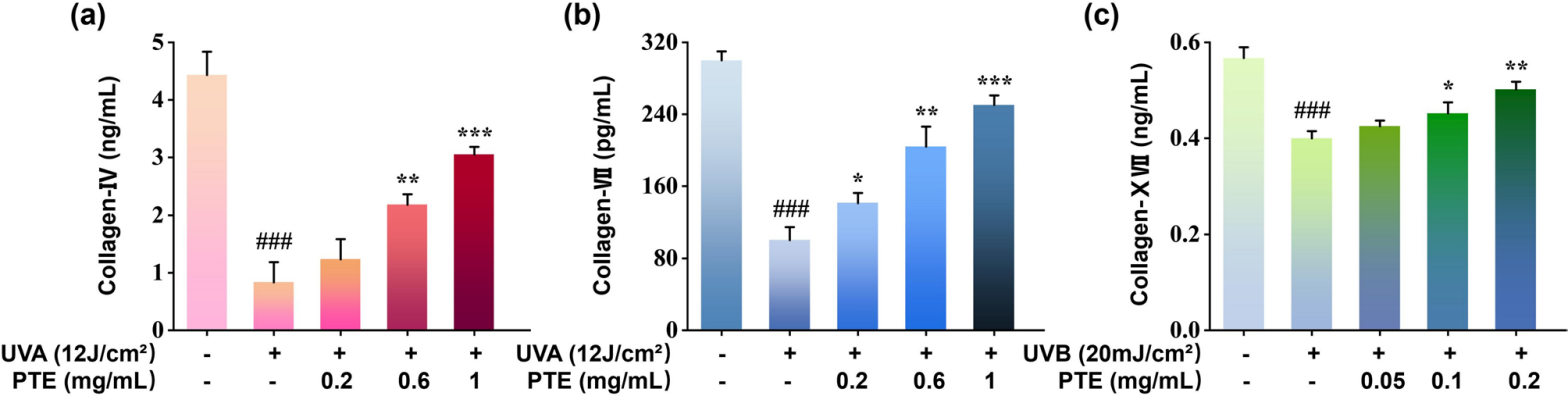
PTE upregulates collagen expression in UV-irradiated skin cells. (**a**‐**b**) Expression of (**a**) Col-IV and (**b**) Col-VII in UVA‐irradiated HDFs. (**c**) Expression of Col-XVII in UVB‐irradiated HaCaT cells. *p < 0.05, **p < 0.01, ***p < 0.001 compared with UV‐irradiated group; #*p* < 0.05, ##*p* < 0.01, ###*p* < 0.001 compared with the blank group.

To investigate the regulatory effect of PTE on collagen metabolism under photodamage conditions, we established two cellular models: HDF cells irradiated with 12 J/cm^2^ UVA and HaCaT cells exposed to 20 mJ/cm^2^ UVB. After 24 h of PTE treatment, the expression levels of Col-IV, VII, and XVII collagen were measured. As illustrated in Fig. 5, UVA irradiation markedly suppressed the expression of type IV and VII collagen in HDF cells, while UVB irradiation significantly reduced type XVII collagen expression in HaCaT cells. Note that collagen XVII is specifically expressed in keratinocytes. Treatment with PTE dose-dependently rescued the UV-suppressed expression of collagens IV, VII, and XVII, suggesting its protective effect against photodegradation. Critically, the restoration of collagens IV and VII, which are key components of the basement membrane (BM)^45^, suggests PTE aids in repairing UV-disrupted BM structure. As BM integrity is fundamental for skin regeneration and proper keratinocyte anchorage and stratification^46,47^, this action supports epidermal repair. Furthermore, by reinforcing the dermal-epidermal junction, PTE may help prevent the downward migration of melanocytes and abnormal melanin persistence, mechanisms linked to chronic hyperpigmentation^48^. Thus, beyond direct melanogenesis inhibition, PTE’s collagen-promoting activity addresses a structural vulnerability in photoaged skin, highlighting its dual benefit in combating both structural aging and dyspigmentation through functional restoration of the dermal-epidermal junction.

### 3.6 inhibitory effect of PTE on UVB-induced melanogenesis

**Fig. 6:**
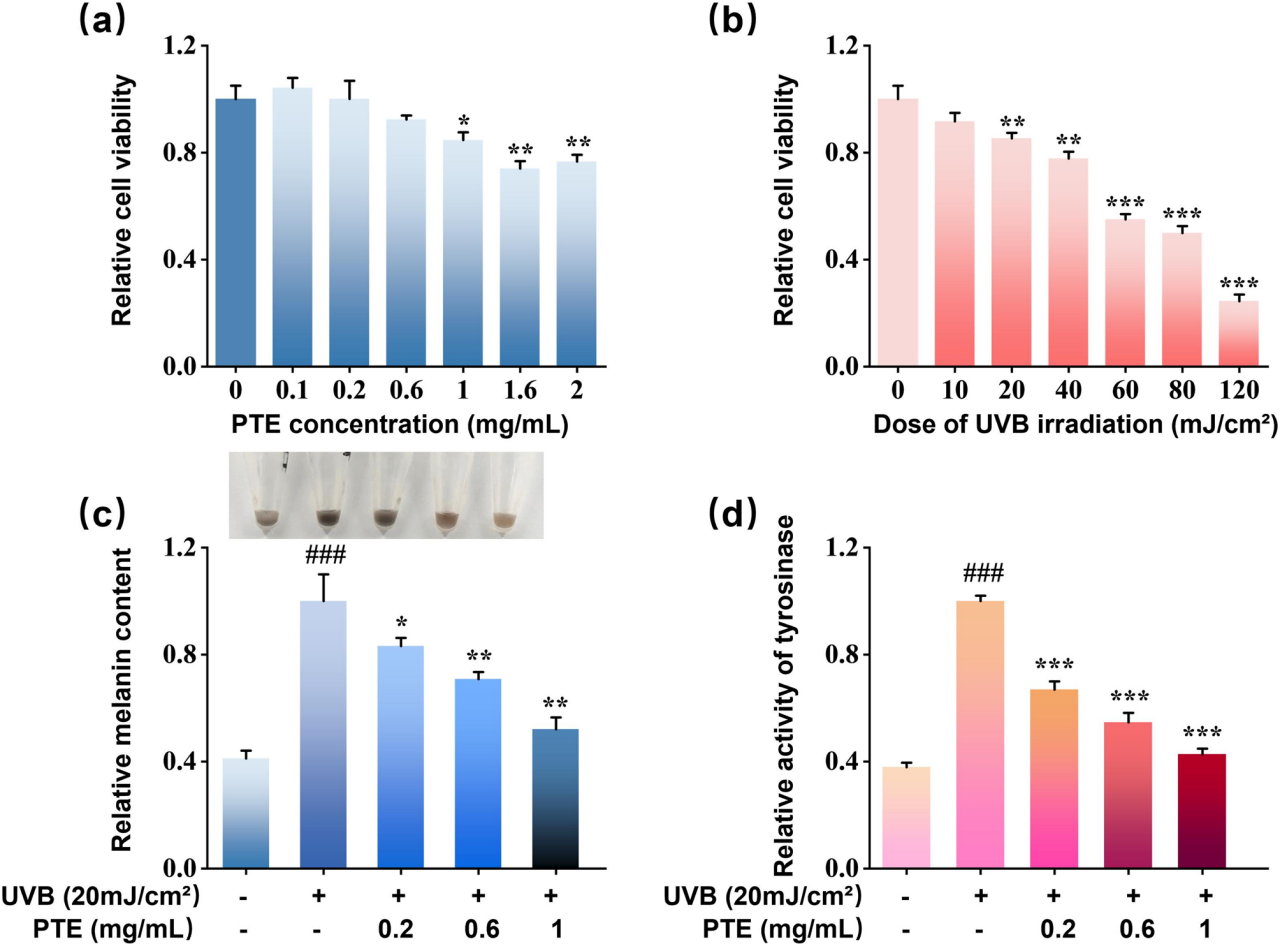
Inhibitory effect of PTE on UVB-induced melanogenesis in B16F10 cells. Cytotoxicity of (**a**) PTE and (**b**) UVB on B16F10 Cells in a dose-dependent manner. (**c**) Measurement of intracellular melanin content. (**d**) Assessment of intracellular TYR activity. *p < 0.05, **p < 0.01, ***p < 0.001 compared with UVB-treated group; #*p* < 0.05, ##*p* < 0.01, ###*p* < 0.001 compared with blank group.

The cytotoxicity of PTE and UVB irradiation on B16F10 cells was evaluated using the MTT assay. As illustrated in Fig. 6a, PTE at concentrations ranging from 0.1 to 1 mg/mL did not significantly affect cell viability. However, a concentration of 2 mg/mL resulted in a pronounced decrease in viability (*p* < 0.05). The non-cytotoxic concentration range of 0.1–1 mg/mL was thus employed in further studies. UVB irradiation exposure resulted in a dose-dependent reduction in cell viability (Fig. 6b). A radiation dose of 20 mJ/cm^2^ was selected for subsequent experiments, as it effectively induced melanogenesis without exerting significant cytotoxic effect. To examine the anti-melanogenic activity of PTE, intracellular melanin content was quantified following UVB exposure. As shown in Fig. 6c, UVB irradiation significantly promoted melanin production compared to the control group (*p* < 0.001), while treatment with PTE dose-dependently attenuated melanin accumulation, which was visually corroborated by the digital images (the inset in Fig. 6c) of the melanocytes cultured under different conditions. Further assessment of TYR activity demonstrated that UVB stimulation markedly enhanced TYR activity (*p* < 0.001), which was dose-dependently suppressed by PTE treatment (*p* < 0.001; Fig. 6d). These findings indicate that PTE likely mitigates UVB-induced melanogenesis through the inhibition of TYR activity.

### 3.7 integrated analysis of transcriptomics and proteomics

**Fig. 7:**
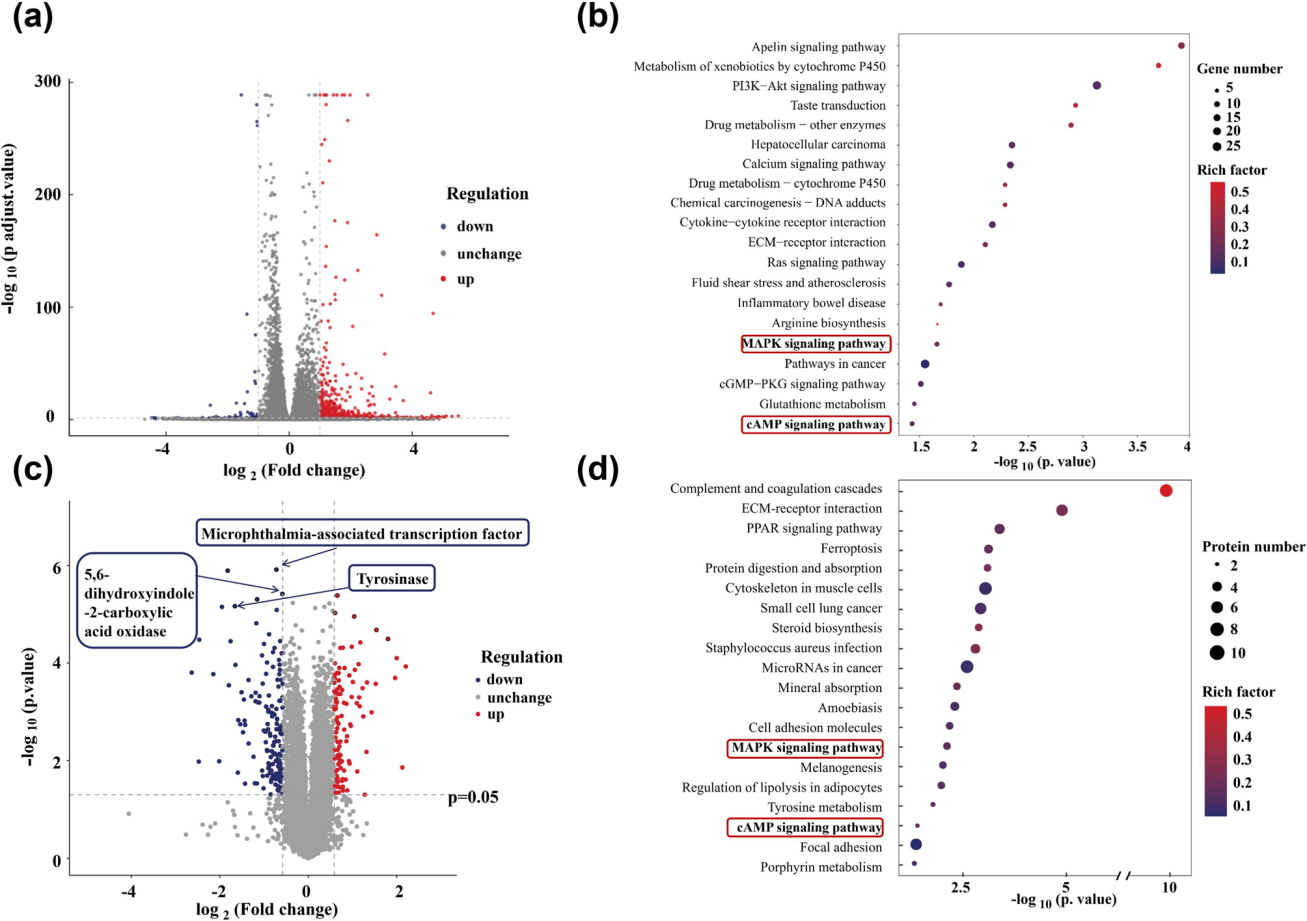
Transcriptome differential gene analysis. (**a**) Differential gene volcano map of model (UVB) versus PTE group. (**b**) KEGG pathway map of DEGs. Proteomics differential proteins analysis. (**c**) Differential protein volcano map of model (UVB) vs. PTE group. (**d**) KEGG pathway map of DEPs.

This study employed an integrated transcriptomic and proteomic approach to systematically elucidate the multi-target mechanism through which PTE inhibits UVB-induced melanogenesis. Transcriptomic profiling identified 729 significantly differentially expressed genes (DEGs), consisting of 668 up-regulated and 61 down-regulated genes (Fig. 7a). Parallel proteomic analysis revealed 260 significantly DEPs, with 111 up-regulated and 149 down-regulated (Fig. 7c). Joint analysis revealed a convergent modulation of the MAPK and cAMP signaling pathways, underscoring their central role in mediating the anti-melanogenic activity of PTE.

KEGG pathway enrichment analysis of the transcriptomic data (Fig. 7b) showed that the DEGs were significantly enriched in multiple pathways related to skin pigmentation regulation, including xenobiotic metabolism by cytochrome P450, phosphatidyqinositol-3 kinase (PI3K-Akt) signaling pathway, MAPK signaling pathway, cyclic guanosine monophosphate-protein kinase G (cGMP-PKG) signaling pathway, glutathione metabolism, and cAMP signaling pathway. These pathways collectively form a complex regulatory network that may underlie the inhibitory effect of PTE on melanogenesis. Consistent with these findings, proteomic-level KEGG analysis (Fig. 7d) indicated significant enrichment of DEPs in the MAPK signaling pathway, melanogenesis, tyrosine metabolism, and cAMP signaling pathway. Notably, key melanogenic proteins such as TYR, 5,6-Dihydroxyindole-2-carboxylic acid oxidase (TYRP1), DCT/TYRP2, and MITF exhibited significant alterations in expression (Fig. 7c). The downregulation of these functionally essential proteins further supports the inhibitory role of PTE in melanogenesis at the molecular level.

In summary, both the MAPK and cAMP signaling pathways were significantly enriched at both the transcriptomic and proteomic levels, highlighting their pivotal role in the regulatory mechanism of PTE. The cAMP signaling pathway modulates CREB phosphorylation, which in turn regulates MITF transcriptional activity^49^. Concurrently, the MAPK pathway, including the ERK, JNK, and p38 sub-pathways influences both the stability and transcriptional activity of MITF through phosphorylation-dependent mechanisms^13–16^. These results suggest that PTE concurrently targets both the cAMP-CREB and MAPK signaling cascades to synergistically suppress MITF expression and activity, ultimately leading to the downregulation of key melanogenic enzymes such as TYR, TYRP1, and TYRP2.

### 3.8 PTE suppresses melanogenic gene expression by targeting cAMP and MAPK signaling pathways

**Fig. 8:**
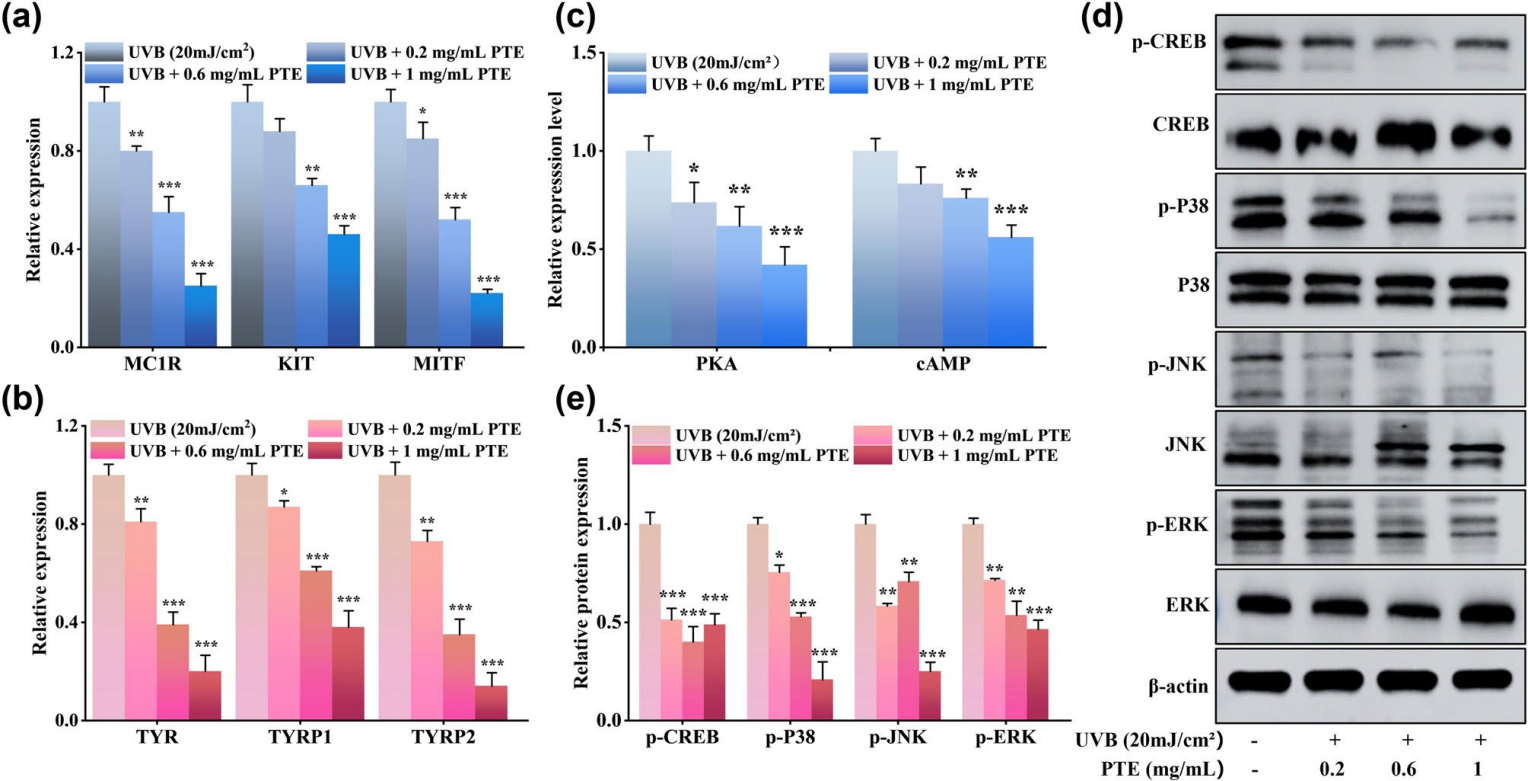
Effect of PTE on the expression levels of melanogenic enzymes (**a**) MC1R, KIT, MITF; (**b**) TYR, TYRP1, TYRP2 in B16F10 cells. (**c**) Intracellular cAMP levels and PKA activity measured using ELISA. (**d**) Representative WB images of p-CREB, CREB, p-p38, P38, p-JNK, JNK, p-ERK, and ERK. (**e**) Quantitative analysis of phosphorylated and total protein levels from (**d**). *p < 0.05, **p < 0.01, ***p < 0.01 compared to the UVB-treated group.

To experimentally validate the molecular mechanism inferred from multi-omics analyses, we performed qPCR and assessed protein phosphorylation levels. The qPCR results indicated that PTE treatment dose-dependently and significantly suppressed the mRNA expression of key melanogenesis-related genes, including MC1R, KIT, MITF, TYR, TYRP1, and TYRP2 (Fig. 8a, b). Notably, inhibition of MITF, which is the master transcriptional regulator of melanogenesis, was most substantial, with mRNA levels reduced by approximately 80% following treatment with 1 mg/mL PTE (*p* < 0.001). These results imply that PTE exerts its anti-melanogenic effect primarily through an MITF-dependent mechanism: by downregulating MITF, PTE subsequently represses the expression of its downstream targets, including TYR, TYRP1 and TYRP2. This observation aligns closely with our proteomic data, which revealed reduced expression of key enzymes such as TYR and DCT/TYRP2.

To elucidate the upstream signaling mechanism by which PTE modulates MITF expression, we conducted WB analyses. UVB radiation activated both cAMP/PKA and MAPK signaling to promote melanogenesis, while PTE significantly reduced intracellular cAMP levels and PKA activity (*p* < 0.001; Fig. 8c). WB results further indicated that PTE markedly suppressed the phosphorylation of CREB and key MAPK members, including p38, ERK, and JNK (*p* < 0.001; Fig. 8d, e). These findings suggest that PTE disrupts MITF regulation through dual inhibition of the cAMP-PKA-CREB and MAPK (p38/ERK/JNK) axes, thereby attenuating both the transcriptional activation and post-translational modification of MITF.

In summary, complementary qPCR and WB analyses strongly support the conclusions derived from dual-omics integration. These results collectively demonstrate that PTE inhibits UVB-induced melanogenesis via concurrent suppression of the cAMP/PKA-CREB and MAPK signaling pathways, leading to reduced MITF transcriptional activity and downregulation of downstream melanogenic genes.

## 4. Discussion

This study provides comprehensive evidence that PTE effectively mitigates UVB-induced melanogenesis through a multi-target mechanism. This action primarily involves the dual suppression of the cAMP/PKA/CREB and MAPK signaling pathways, culminating in the downregulation of MITF and its downstream melanogenic enzymes. Furthermore, PTE demonstrates significant anti-inflammatory and collagen-stabilizing activities, highlighting its potential as a multi-functional natural agent for managing hyperpigmentation while supporting overall skin barrier integrity.

The anti-melanogenic effect of PTE operates through a coordinated intervention at multiple biological levels. Initially, PTE alleviates the UVB-induced inflammatory response in keratinocytes. This anti-inflammatory action is supported by its capacity to attenuate upstream oxidative stress, as directly evidenced by the dose-dependent reduction in intracellular ROS generation—a finding consistent with network pharmacology predictions implicating key antioxidant regulators such as NFE2L2 and SOD1. The mitigation of inflammatory and oxidative stress subsequently leads to a decreased secretion of critical paracrine melanogenic factors (including α-MSH, bFGF, and ET-1) from keratinocytes, thereby reducing the activation stimulus for neighboring melanocytes. Subsequently, within melanocytes, our integrated transcriptomic and proteomic analyses convergently identified the cAMP/PKA/CREB and MAPK pathways as central hubs targeted by PTE. This systems-level insight was functionally validated: PTE significantly reduced intracellular cAMP levels and PKA activity, while also inhibiting the phosphorylation of CREB and key MAPK members (p38, ERK, and JNK). The resultant downregulation of both MITF mRNA and protein expression, followed by the suppression of TYR, TYRP1, and TYRP2, delineates a coherent causal chain from signal transduction inhibition to the final phenotypic output of reduced melanin synthesis. This multi-target approach is particularly advantageous, as it likely prevents compensatory activation within the highly interconnected melanogenic network, potentially offering more robust efficacy compared to single-target agents.

Beyond direct melanogenesis inhibition, PTE’s anti-inflammatory and collagen-stabilizing properties contribute synergistically to its overall skin-lightening potential. By suppressing UVB-induced inflammatory cytokines (such as IL-1α, IL-6, PGE2, and TNF-α) in keratinocytes, PTE addresses a primary trigger of post-inflammatory PIH. Moreover, its ability to counteract UV-induced degradation of collagens IV, VII, and XVII suggests a protective role in maintaining basement membrane integrity. A stable dermal-epidermal junction is crucial for normal pigmentary homeostasis, preventing the downward migration of melanocytes and the subsequent formation of persistent pigmentation. Therefore, PTE not only directly inhibits melanin production but also addresses fundamental inflammatory and structural vulnerabilities implicated in pigmentary disorders.

## Conclusion

In conclusion, this study elucidates the multi-target anti-melanogenic mechanism of PTE through an integrated approach combining network pharmacology, transcriptomics, proteomics, and in vitro assays. PTE alleviates UVB-induced oxidative stress by reducing ROS generation, which underlies its anti-inflammatory effect and the subsequent reduction in pro-melanogenic paracrine factor secretion from keratinocytes. Within melanocytes, PTE concurrently suppresses the cAMP/PKA/CREB and MAPK signaling pathways, downregulating MITF and its downstream enzymes (TYR, TYRP1, TYRP2). Additionally, PTE mitigates UV-induced collagen degradation, thereby supporting dermal-epidermal junction integrity.

These findings indicate that PTE possesses not only direct melanogenesis-inhibitory activity but also anti-inflammatory, antioxidant, and photo-damage repair functions. This multifaceted and coordinated mechanism, revealed for the first time through an integrated systems-level approach, positions PTE as a promising natural candidate for managing hyperpigmentation disorders. By employing a systems-level strategy integrating pharmacology and multi-omics, this work provides a deeper understanding of the complex polypharmacological network of botanical extracts. It contributes to the development of natural, multi-target alternatives in skincare, aligning with the growing trend towards clean beauty and sustainable cosmetic formulations. Future research should focus on the isolation and identification of PTE’s key active constituents, in vivo validation of its efficacy and safety, and the development of effective topical delivery systems to translate these mechanistic insights into practical applications.

## Data Availability

Data is provided within the manuscript or supplementary information files. Data from transcriptomics experiments have been deposited in NCBI’s Gene Expression Omnibus. The previously generated datasets, that are already published, are accessible through GEO Series accession number GSE315802. The mass spectrometry proteomics data have been deposited to the ProteomeXchange Consortium via the PRIDE partner repository with the dataset identifier PXD072752.

## Supplementary Information

Below is the link to the electronic supplementary material.

Supplementary Material 1

## Data Availability

Data is provided within the manuscript or supplementary information files. Data from transcriptomics experiments have been deposited in NCBI’s Gene Expression Omnibus. The previously generated datasets, that are already published, are accessible through GEO Series accession number GSE315802. The mass spectrometry proteomics data have been deposited to the ProteomeXchange Consortium via the PRIDE partner repository with the dataset identifier PXD072752.
