# Supplementary material for: Polianthes tuberosa L. Extract suppresses melanogenesis through concurrent Inhibition of cAMP/CREB and MAPK signaling pathways
Source: Sci Rep. 2026 Jan 24;16:6137. doi: 10.1038/s41598-026-36962-9 (PMC12901037; doi:10.1038/s41598-026-36962-9)
Supplement: Supplementary file 1 — Supplementary Material 1 [file 41598_2026_36962_MOESM1_ESM.docx]

**Anti-Melanogenesis Mechanism of *Polianthes tuberosa L*. Extract through Network Pharmacology and Dual-Omics: Multi-Target Inhibition via cAMP/CREB and MAPK Pathways**

Qiaozhen Li,^1^ Hui Zhu,^2^ Teng Jiang,^2^ Rubiao Hou,^2^ Jinhua Li,^1,3^ Xiaodong Yan,^1, *^ Jing Wang^1, *^

1. Key Laboratory of Synthetic and Biological Colloids, Ministry of Education, School of Chemical & Material Engineering, Jiangnan University, Wuxi, Jiangsu, 214122, China

2. Brightday and Dream Biotechnology Co., Ltd., Hangzhou, Zhejiang, 310015, China

3. Jiangnan Institute of Beauty Research, Wuxi 214111, China

*Email: [xiaodong.yan@jiangnan.edu.cn](mailto:xiaodong.yan@jiangnan.edu.cn); [jingwang@jiangnan.edu.cn](mailto:jingwang@jiangnan.edu.cn)

Table S1 Identification of the chemical constituents of PTE using UPLC-HRMS.

| Peak name | m/z | Retention time (min) | ppm | compound name | adduct | score | Pubchem number |
| --- | --- | --- | --- | --- | --- | --- | --- |
| 1 | 104.1074 | 1.58 | 2.6 | 2-(Dimethylamino)propanol | [M+H]^+^ | 0.9992 | 85865 |
| 2 | 118.0865 | 1.68 | 1.1 | Betaine | [M+H]^+^ | 0.9961 | 247 |
| 3 | 130.05 | 1.99 | 0.5 | O-Acetyl-L-serine | [M+H−H_2_O]^+^ | 0.9977 | 189 |
| 4 | 268.1038 | 2.03 | 0 | Adenosine | [M+H]^+^ | 0.9997 | 447270 |
| 5 | 132.1021 | 2.15 | 0.3 | Triethylolamine | [M+H−H_2_O]^+^ | 0.9785 | 7618 |
| 6 | 166.0863 | 2.8 | 0.5 | Valylphenylalanine | [M+H−C_5_H_9_NO]^+^ | 0.9995 | 144746 |
| 7 | 205.0972 | 3.47 | 0.9 | L-Tryptophan | [M+H]^+^ | 0.9976 | 6305 |
| 8 | 287.0547 | 5.22 | 1.9 | Kaempferol | [M+H]^+^ | 0.9998 | 5280863 |
| 9 | 275.2004 | 9.43 | 0.6 | 9,10,11-Trihydroxy-12(Z),15(Z)-octadecadienoic acid | [M+H−3H_2_O]^+^ | 0.9736 | 56671117 |
| 10 | 447.3104 | 11.2 | 0.2 | Ecdysone | [M+H−H_2_O]^+^ | 0.8522 | 5316996 |
| 11 | 149.0233 | 14.18 | 0.4 | Di(2,6-dimethyl-4-heptyl) phthalate | [M+H−C_18_H_38_O]^+^ | 0.9986 | 57003681 |
| 12 | 282.2791 | 15.78 | 0.4 | Oleamide | [M+H]^+^ | 0.9526 | 5283387 |
| 13 | 191.019 | 1.67 | 2.5 | Citric acid | [M−H]^−^ | 0.974 | 311 |
| 14 | 117.0186 | 2.15 | 7 | Succinic acid | [M−H]^−^ | 0.9099 | 1110 |
| 15 | 164.0711 | 2.79 | 3.7 | N-Acetyl-D-phenylalanine | [M−H−C_2_H_2_O]^−^ | 0.9833 | 101184 |
| 16 | 355.0673 | 3.38 | 4.9 | Coumaroyl + c6h9o8 (isomer of 844, 845, 846) | [M−H]^−^ | 0.8502 | 145994453 |
| 17 | 239.056 | 4.05 | 0.1 | Acetylsyringic acid | [M−H]^−^ | 0.7794 | 225680 |
| 18 | 210.0769 | 4.33 | 1.5 | 3-Methoxytyrosine | [M−H]^−^ | 0.9542 | 1670 |
| 19 | 461.1672 | 4.39 | 1.2 | Hydrageifolin I | [M+HCOO]^−^ | 0.9785 | 10549806 |
| 20 | 741.1906 | 4.58 | 3.3 | 2-(3,4-Dihydroxyphenyl)-5,7-dihydroxy-4-oxo-4H-chromen-3-yl 6-O-(6-deoxy-2-O-((2S,3R,4R)-3,4-dihydroxy-4-(hydroxymethyl)tetrahydrofuran-2-yl)-.alpha.-L-mannopyranosyl)-.beta.-D-glucopyranoside | [M−H]^−^ | 0.9782 |  |
| 21 | 241.1194 | 4.62 | 0.6 | L-.gamma.-Glutamyl-L-leucine | [M−H−H_2_O]^−^ | 0.9926 | 4524287 |
| 22 | 595.1317 | 4.81 | 1.9 | Quercetin-3-O-.beta.-D-xylopyranosyl (1->6)-.beta.-D-glucopyranoside | [M−H]^−^ | 0.9984 | 5315208 |
| 23 | 725.195 | 4.93 | 1.4 | Camelliaside B | [M−H]^−^ | 0.9213 | 25115190 |
| 24 | 579.1361 | 5.22 | 0.5 | Leucoside | [M−H]^−^ | 0.9851 | 44566720 |
| 25 | 593.1523 | 5.58 | 2.2 | Kaempferol 3-rungioside | [M−H]^−^ | 0.9911 | 23815431 |
| 26 | 187.0967 | 6.48 | 2.5 | Azelaic acid | [M−H]^−^ | 0.9956 | 2266 |
| 27 | 327.2177 | 9.42 | 0.1 | (10E,15Z)-9,12,13-Trihydroxyoctadeca-10,15-dienoic acid | [M−H]− | 0.9983 | 44559173 |
| 28 | 329.2333 | 10.25 | 6.4 | FA 18:1+3o | [M−H]^−^ | 0.9988 | 153001 |


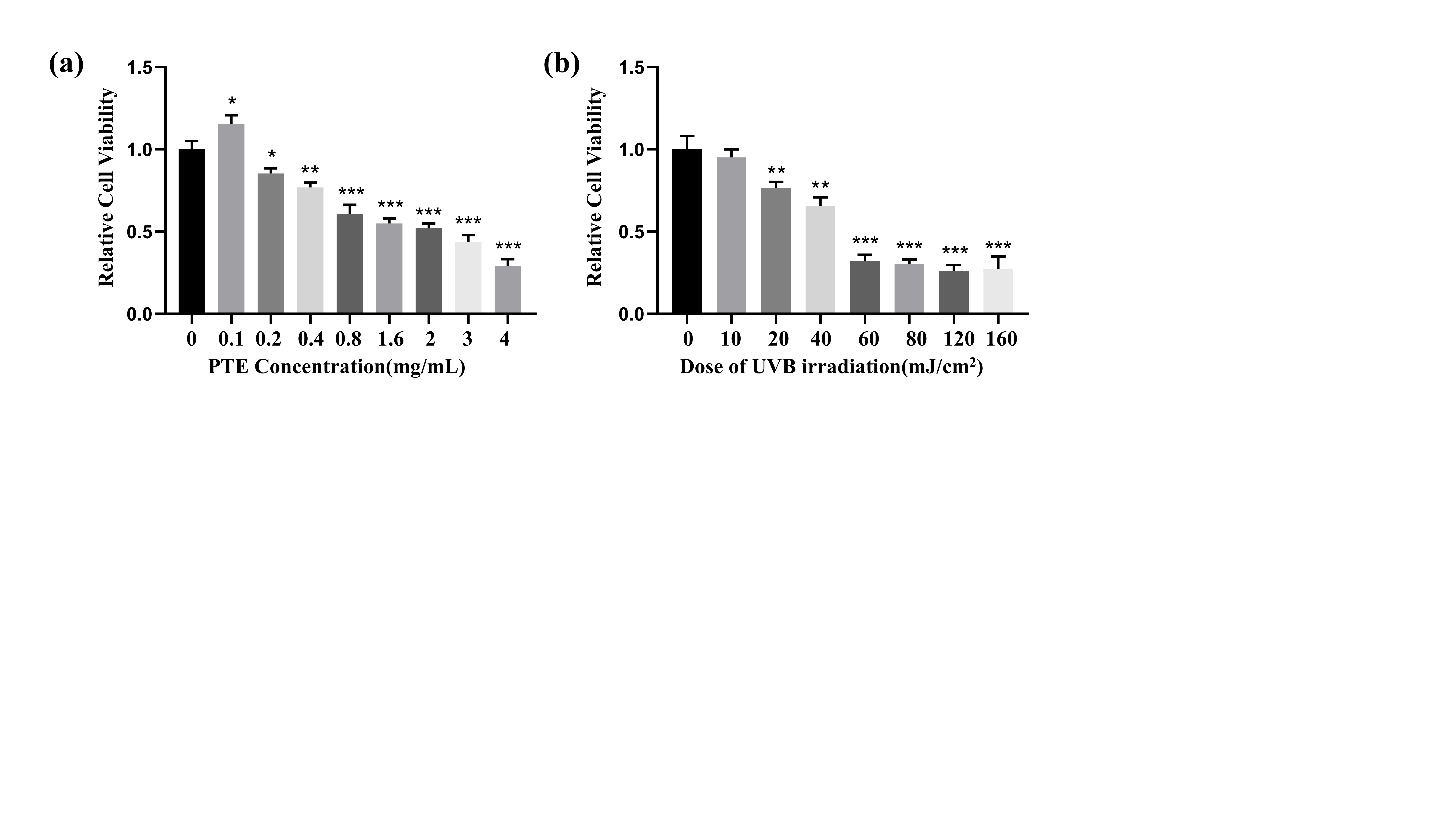


Figure S1. (a) Cytotoxic Effects of Different PTE concentration on HaCat Cells Measured by MTT Assay. (b) Cytotoxic Effects of Different UVB Doses PTE concentration on HaCat Cells Measured by MTT


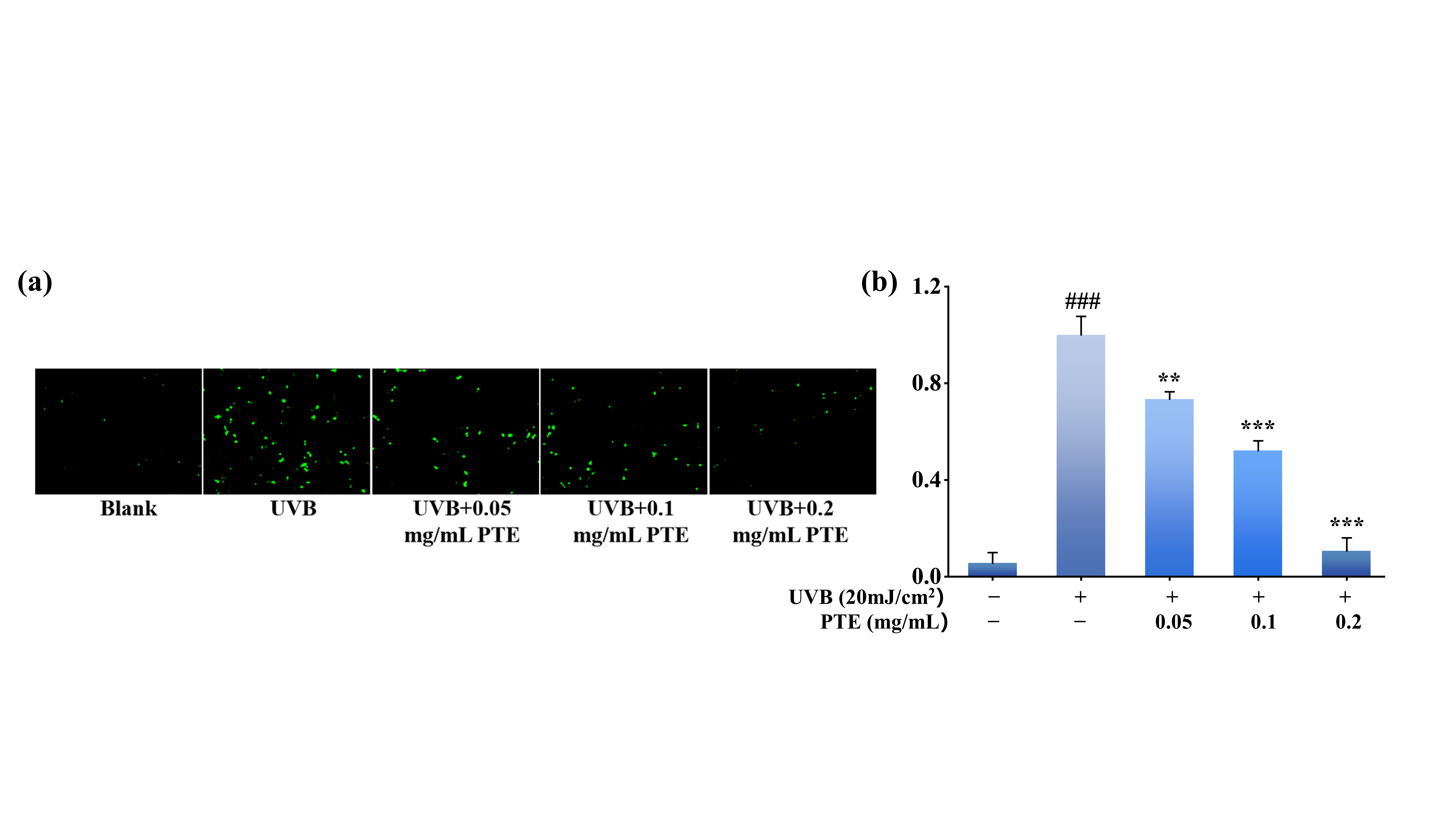


Figure S2. (a) Representative fluorescence micrographs of intracellular ROS levels detected by DCFH-DA probe following 20 mJ/cm^2^ UVB irradiation. (b) Quantification of DCF fluorescence intensity.


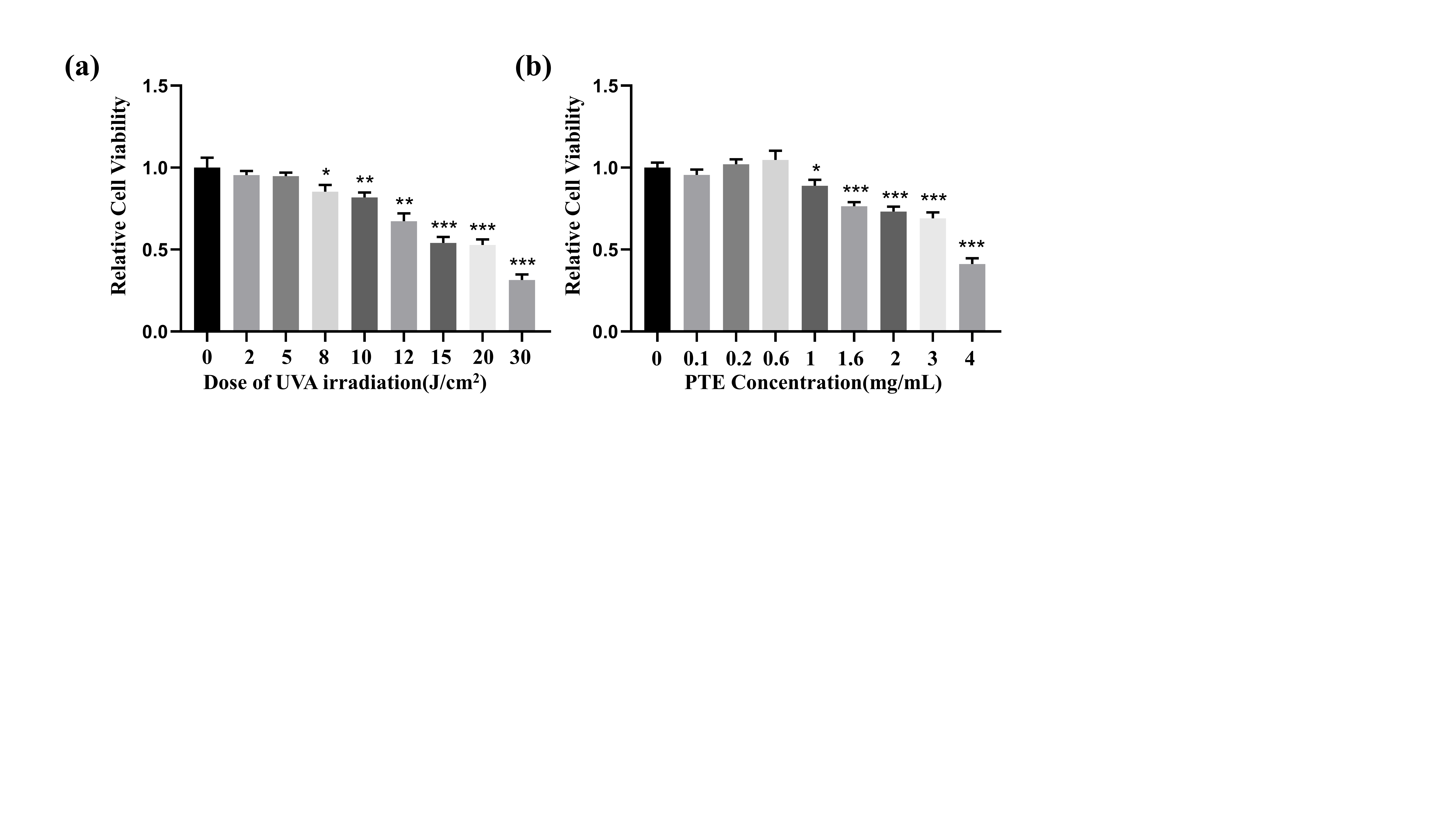


Figure S3. (a) Cytotoxic Effects of Different UVA Doses on HDF Cells Measured by MTT Assay. (b) Cytotoxic Effects of Different PTE concentration PTE concentration on HDF Cells Measured by MTT Assay.
